# Quasi-Concavity for Gaussian Multicast Relay Channels

**DOI:** 10.3390/e21020109

**Published:** 2019-01-24

**Authors:** Mohit Thakur, Gerhard Kramer

**Affiliations:** 1 Independent Researcher, Amalienstr. 49A, 80799 Munich, Germany; 2 Institute for Communications Engineering, Technical University of Munich, 80333 Munich, Germany

**Keywords:** capacity, decode-forward, multicast, relaying

## Abstract

Standard upper and lower bounds on the capacity of relay channels are cut-set (CS), decode-forward (DF), and quantize-forward (QF) rates. For real additive white Gaussian noise (AWGN) multicast relay channels with one source node and one relay node, these bounds are shown to be quasi-concave in the receiver signal-to-noise ratios and the squared source-relay correlation coefficient. Furthermore, the CS rates are shown to be quasi-concave in the relay position for a fixed correlation coefficient, and the DF rates are shown to be quasi-concave in the relay position. The latter property characterizes the optimal relay position when using DF. The results extend to complex AWGN channels with random phase variations.

## 1. Introduction

A multicast relay channel (MRC) is an information network with a source node, a relay node, and two or more destination nodes, and where one message originating at the source should be received reliably at the destinations. We consider additive white Gaussian noise (AWGN) MRCs and show that certain information rate expressions are quasi-concave in the receiver signal-to-noise ratios (SNRs), the squared source-relay correlation coefficient, and the relay position. In particular, we study cut-set (CS), decode-forward (DF), and quantize-forward (QF) rates. Quasi-concavity suggests that efficient algorithms can optimize signaling and the relay position. However, the main motivation of this work is not practicality, but simply to provide better understanding of the problem.

Relay positioning has been studied by many authors, with a focus on rate enhancement (e.g., [1,2]), range extension (e.g., [3,4]), and outage probability (e.g., [1,5,6]). We study the problem of placing a relay to maximize the multicast rate by extending results of [7,8,9,10]. A preliminary version of this paper without proofs appeared in [11]. Our focus is on real alphabet channels. However, our main results also apply to complex alphabet channels if there are random phase variations so that beamforming is not useful.

This paper is organized as follows. Section 2 presents the MRC model and reviews the CS, DF, and QF rates. Section 3 develops quasi-concavity results in the squared source-relay correlation coefficient $\rho^{2}$ and the channel SNRs. Section 4 introduces a distance dependence for the channel gains and shows that the CS rate is quasi-concave in the relay position when ρ is fixed. We further show that the DF rate is quasi-concave in the relay position. Section 5 illustrates quasi-concavity for one-, two-, and three-dimensional networks, and compares the performance of two DF strategies. Section 6 discusses complex AWGN channels and a sum (source plus relay) power constraint. Section 7 concludes the paper. Appendix A and Appendix B review useful results on concavity and quasi-concavity, and prove a few new results.

## 2. Model and Information Rates

### 2.1. Model

An MRC has three types of nodes:

- a source node *s* that generates a message *W* and transmits the symbols $X_{s}^{n}=X_{s,1},X_{s,2},\ldots ,X_{s,n}$;
- a relay node *r* that receives and forwards symbols $Y_{r,k}$ and $X_{r,k}$, respectively, for k=1,2,⋯,n;
- destination nodes j=1,2,…,N where node *j* receives $Y_{j}^{n}=Y_{j,1},Y_{j,2},\ldots ,Y_{j,n}$ and estimates *W* as ${\hat{W}}_{j}$.

**Figure 1:**
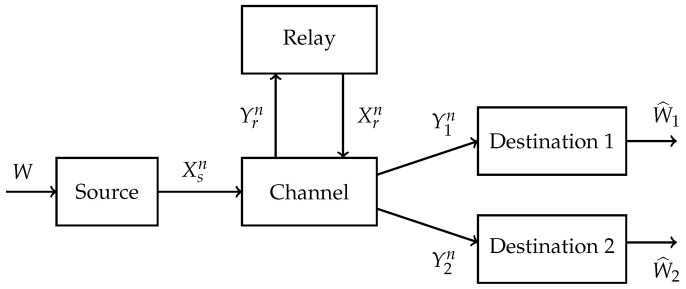
Multicast relay channel (MRC) with two destinations.

We denote the destination node set as T={1,2,…,N}. The classic relay channel has N=1 and Figure 1 shows an MRC with N=2.

A memoryless MRC has a function h(·) and a noise random variable Z so that for every time instant the N+1 channel outputs $Y=(Y_{r}\;Y_{1}\;\ldots \;Y_{N})$ are given by

$$\begin{array}{c} Y=h(X_{s},X_{r},Z). \end{array}$$

The noise Z is statistically independent of $X_{s}$ and $X_{r}$, and the noise variables at different times are statistically independent.

An encoding strategy for *M* messages has

- *W* uniformly distributed over {1,2,…,M};
- an encoding function $e_{s}\left(\cdot \right)$ such that $X_{s}^{n}=e_{s}\left(W\right)$;
- relay functions $e_{r,k}\left(\cdot \right)$ with $X_{r,k}=e_{r,k}\left(Y_{r,1},\ldots ,Y_{r,k-1}\right)$, where k=1,2,…,n;
- decoding functions $d_{j}\left(\cdot \right)$ such that $d_{j}\left(Y_{j}^{n}\right)={\hat{W}}_{j}$, j∈T.

The error probability at destination *j* is $P_{e,j}=\mathrm{Pr}\left[{\hat{W}}_{j}\neq W\right]$. The multicast rate is $R=(\log_{2}M)/n$ bits/use. The rate *R* is achievable if, for any ϵ>0 and sufficiently large *n*, there is an encoding strategy with $P_{e,j}\leq \epsilon$ for all j∈T. The capacity *C* is the supremum of the achievable rates.

### 2.2. Information Rates

The following bounds were given in [12] for the relay channel (N=1). Their extensions to MRCs are straightforward.

- CS Rate: $C\leq R_{CS}$ where
  (1) $$\begin{array}{cc} R_{CS}= & \max\left\{\min_{1\leq j\leq N}\min\left(I\left(X_{s}X_{r};Y_{j}\right),I\left(X_{s};Y_{r}Y_{j}|X_{r}\right)\right)\right\} \end{array}$$
  and where the maximization is over all $X_{s}X_{r}$.
- Direct-Transmission (DT) Rate: $C\geq R_{DT}$ where
  (2) $$\begin{array}{c} R_{DT}=\max\left\{\min_{1\leq j\leq N}I\left(X_{s};Y_{j}|X_{r}=x^{*}\right)\right\} \end{array}$$
  and where the maximization is over all $x^{*}$ and $X_{s}$.
- DF Rate: $C\geq R_{DF}$ where
  (3) $$\begin{array}{cc} R_{DF}= & \max\left\{\min_{1\leq j\leq N}\min\left(I\left(X_{s}X_{r};Y_{j}\right),I\left(X_{s};Y_{r}|X_{r}\right)\right)\right\} \end{array}$$
  and where the maximization is over all $X_{s}X_{r}$.
- QF Rate: $C\geq R_{QF}$ where
  (4) $$\begin{array}{cc} R_{QF}= & \max\left\{\min_{1\leq j\leq N}\min\left(I\left(X_{s}X_{r};Y_{j}\right)-I\left(Y_{r};{\hat{Y}}_{r}|X_{s}X_{r}Y_{j}\right),I\left(X_{s};{\hat{Y}}_{r}Y_{j}|X_{r}\right)\right)\right\} \end{array}$$
  where ${\hat{Y}}_{r}$ is an auxiliary random variable, and where the maximization is over all $X_{s}X_{r}{\hat{Y}}_{r}$ such that $X_{s}$ and $X_{r}$ are independent and $X_{s}-X_{r}Y_{r}-{\hat{Y}}_{r}$ forms a Markov chain.

### 2.3. Real Alphabet AWGN MRC

**Figure 2:**
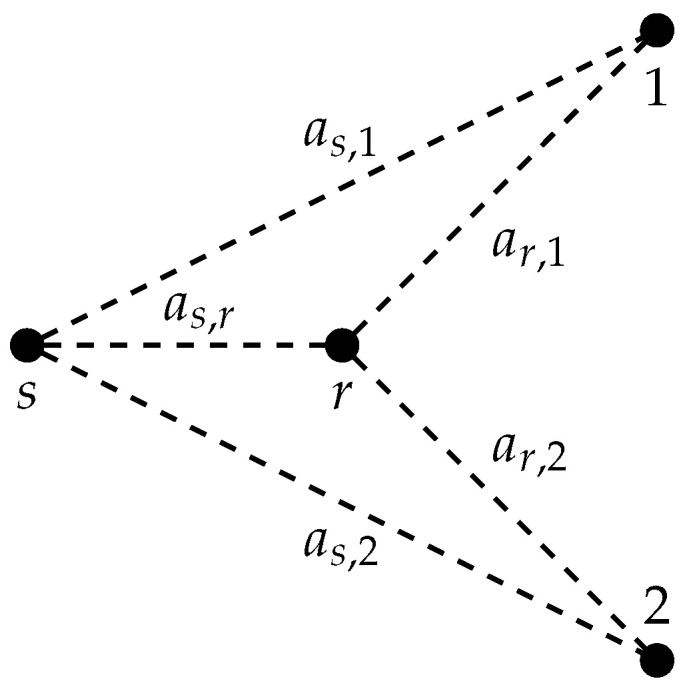
AWGN MRC with two destinations.

The real alphabet AWGN MRC has real channel symbols and

(5) $$\begin{array}{cc} Y_{r} & =a_{s,r}X_{s}+Z_{r} \end{array}$$

(6) $$\begin{array}{cc} Y_{j} & =a_{s,j}X_{s}+a_{r,j}X_{r}+Z_{j} \end{array}$$

where j∈T. The $a_{s,r}$, $a_{s,j}$, and $a_{r,j}$ are channel gains between the nodes (see Figure 2). We later relate these gains to distances between the nodes. The $Z_{r}$ and $Z_{j}$, j=1,2,…,N, are independent and identically distributed Gaussian random variables with zero mean and unit variance. We may alternatively write (5) and (6) in vector form as

(7) $$\begin{array}{c} Y_{j}=A_{j}X+Z_{j} \end{array}$$

where $X={\left(X_{s}\;X_{r}\right)}^{T}$, $Y_{j}={\left(Y_{r}\;Y_{j}\right)}^{T}$, $Z={\left(Z_{r}\;Z_{j}\right)}^{T}$, and

(8) $$\begin{array}{c} A_{j}=\left(\begin{array}{cc} a_{s,r} & 0\\ a_{s,j} & a_{r,j} \end{array}\right). \end{array}$$

We consider individual average block power constraints

(9) $$\begin{array}{c} E\left[\sum_{k=1}^{n}X_{s,k}^{2}\right]\leq nP_{s},\;E\left[\sum_{k=1}^{n}X_{r,k}^{2}\right]\leq nP_{r}. \end{array}$$

The SNR and the capacity of the link from node *u* (with transmit power $P_{u}$) to node *v* are the respective

(10) $$\begin{array}{c} {\mathrm{SNR}}_{u,v}=a_{u,v}^{2}P_{u} \end{array}$$

(11) $$\begin{array}{c} C\left({\mathrm{SNR}}_{u,v}\right)=\frac{1}{2}\log\left(1+{\mathrm{SNR}}_{u,v}\right). \end{array}$$

We simplify the above rate bounds for the AWGN MRC.

- CS Rate:
  (12) $$\begin{array}{cc} R_{CS} & =\max_{\rho }[\min_{1\leq j\leq N}\min\left(C\left({\mathrm{SNR}}_{s,j}+{\mathrm{SNR}}_{r,j}+2\rho \sqrt{{\mathrm{SNR}}_{s,j}{\mathrm{SNR}}_{r,j}}\right),\right.\\ & \;C\left(\left(1-\rho^{2}\right)\left({\mathrm{SNR}}_{s,j}+{\mathrm{SNR}}_{s,r}\right))\right)] \end{array}$$
  where the correlation coefficient ρ satisfies |ρ|≤1. One can restrict attention to non-negative ρ.
- DT Rate:
  (13) $$\begin{array}{c} R_{DT}=\min_{1\leq j\leq N}C\left({\mathrm{SNR}}_{s,j}\right). \end{array}$$
- DF Rate:
  (14) $$\begin{array}{cc} & R_{DF}=\max_{\rho }\left[\min_{1\leq j\leq N}\min\left(C\left({\mathrm{SNR}}_{s,j}+{\mathrm{SNR}}_{r,j}+2\rho \sqrt{{\mathrm{SNR}}_{s,j}{\mathrm{SNR}}_{r,j}}\right),C\left(\left(1-\rho^{2}\right){\mathrm{SNR}}_{s,r}\right)\right)\right]. \end{array}$$
  One can again restrict attention to non-negative ρ.
- QF Rate: Optimizing $X_{s}X_{r}{\hat{Y}}_{r}$ seems difficult. Instead, we choose $X_{s}$ and $X_{r}$ to be zero-mean Gaussian with variances $P_{s}$ and $P_{r}$, respectively. We further choose ${\hat{Y}}_{r}=Y_{r}+Z_{r}$ where $Z_{r}$ is zero-mean Gaussian with variance $N_{r}$. Optimizing $N_{r}$ gives (see [13], pp. 336–337)
  (15) $$\begin{array}{cc} {\tilde{R}}_{QF}= & \min_{1\leq j\leq N}C\left({\mathrm{SNR}}_{s,j}+\frac{{\mathrm{SNR}}_{r,j}{\mathrm{SNR}}_{s,r}}{{\mathrm{SNR}}_{s,j}+{\mathrm{SNR}}_{r,j}+{\mathrm{SNR}}_{s,r}+1}\right). \end{array}$$

## 3. Quasi-Concavity in SNRs and $\rho^{2}$

### 3.1. CS Rate

We consider two characterizations of $R_{CS}$. First, let $a_{j}^{T}=\left(a_{s,j}\;a_{r,j}\right)$ be the second row of $A_{j}$, let $Q_{X}$ be the covariance matrix of X (see Appendix A), and let detM be the determinant of the square matrix M. The CS rate (12) can be expressed as the maximum of

(16) $$\begin{array}{cc} R_{CS}\left(Q_{X}\right)= & \min_{1\leq j\leq N}\min\left(\frac{1}{2}\log\left(a_{j}^{T}\;Q_{X}\;a_{j}+1\right),\frac{1}{2}\log\left(\frac{\det Q_{{\left(Y_{j}^{T}\;X_{r}\right)}^{T}}}{P_{r}}\right)\right) \end{array}$$

over the convex set of $Q_{X}$ with diagonal entries $P_{s}$ and $P_{r}$. The first logarithm in (16) is clearly concave in $Q_{X}$. The second logarithm is concave in $Q_{{\left(Y_{j}^{T}\;X_{r}\right)}^{T}}$ (see Appendix A) and $Q_{{\left(Y_{j}^{T}\;X_{r}\right)}^{T}}$ is linear in $Q_{X}$. To prove the latter claim, observe that

(17) $$\begin{array}{c} Q_{{\left(Y_{j}^{T}\;X_{r}\right)}^{T}}={\tilde{A}}_{j}Q_{X}{\tilde{A}}_{j}^{T}+\left(\begin{array}{cc} I_{2} & 0\\ 0 & 0 \end{array}\right) \end{array}$$

where ${\tilde{A}}_{j}^{T}=\left(A_{j}^{T}\;{\left[0\;1\right]}^{T}\right)$ and $I_{2}$ is the 2×2 identity matrix. Hence $R_{CS}\left(Q_{X}\right)$ is concave in (the convex set of) $Q_{X}$ because it is the minimum of 2N concave functions.

Suppose next that we wish to consider ρ and the SNRs individually rather than via $Q_{X}$. Define the vector

(18) $$\begin{array}{cc} S & =({\mathrm{SNR}}_{s,r},{\mathrm{SNR}}_{s,1},\cdots ,{\mathrm{SNR}}_{s,N},{\mathrm{SNR}}_{r,1},\cdots ,{\mathrm{SNR}}_{r,N}) \end{array}$$

and the functions

(19) $$\begin{array}{c} f_{j}\left(\rho ,S\right)={\mathrm{SNR}}_{s,j}+{\mathrm{SNR}}_{r,j}+2\rho \sqrt{{\mathrm{SNR}}_{s,j}{\mathrm{SNR}}_{r,j}} \end{array}$$

(20) $$\begin{array}{c} g_{j}\left(\rho ,S\right)=\left(1-\rho^{2}\right)\left({\mathrm{SNR}}_{s,j}+{\mathrm{SNR}}_{s,r}\right) \end{array}$$

(21) $$\begin{array}{c} R_{CS}\left(\rho ,S\right)=\min_{1\leq j\leq N}\min\left(C\left(f_{j}\left(\rho ,S\right)\right),C\left(g_{j}\left(\rho ,S\right)\right)\right). \end{array}$$

We establish the following results. We restrict attention to 0≤ρ≤1 and positive S.

**Lemma** **1.** *$f_{j}\left(\rho ,S\right)$ and $g_{j}\left(\rho ,S\right)$ are concave in ρ, concave in S, and quasi-concave in $\left(\rho^{2},S\right)$.*

**Proof.**  Concavity with respect to ρ is established by observing that $f_{j}\left(\rho ,S\right)$ is linear in ρ, and $g_{j}\left(\rho ,S\right)$ is linear in $-\rho^{2}$ which is concave in ρ.

Consider next concavity with respect to S. The Hessian of $f_{j}\left(\rho ,S\right)$ with respect to S has only one non-zero eigenvalue

(22) $$\begin{array}{c} -\frac{\rho }{2}\cdot \frac{{\mathrm{SNR}}_{s,j}^{2}+{\mathrm{SNR}}_{r,j}^{2}}{{\mathrm{SNR}}_{s,j}^{3/2}{\mathrm{SNR}}_{r,j}^{3/2}}. \end{array}$$

Thus, $f_{j}\left(\rho ,S\right)$ is concave in S for non-negative ρ and positive S. The function $g_{j}\left(\rho ,S\right)$ is linear in S, and thus concave in S.

Now consider quasi-concavity with respect to $\left(\rho^{2},S\right)$. Substituting $a={\mathrm{SNR}}_{s,j},b={\mathrm{SNR}}_{r,j},c=\rho^{2}$ into the fifth function of Lemma A6 in Appendix B, we find that $f_{j}\left(\rho ,S\right)$ is quasi-concave in $\left(\rho^{2},S\right)$. For the $g_{j}\left(\rho ,S\right)$, observe that ab is quasi-concave for non-negative (a,b), see the first function of Lemma A6. This implies (see (A7))

(23) $$\begin{array}{c} \left(\lambda a_{1}+\bar{\lambda }a_{2}\right)\left(\lambda b_{1}+\bar{\lambda }b_{2}\right)\geq \min\left(a_{1}b_{1},a_{2}b_{2}\right) \end{array}$$

for 0≤λ≤1, and where $\bar{\lambda }=1-\lambda$. Substituting $a_{i}=1-\rho_{i}^{2}$ and $b_{i}={\mathrm{SNR}}_{s,j,i}+{\mathrm{SNR}}_{s,r,i}$ for i=1,2, we find that $g_{j}\left(\rho ,S\right)$ is quasi-concave in $\left(\rho^{2},S\right)$. ☐

**Theorem** **1.** *$R_{CS}\left(\rho ,S\right)$ is concave in ρ, concave in S, and quasi-concave in $\left(\rho^{2},S\right)$.*

**Proof.**  $R_{CS}\left(\rho ,S\right)$ involves taking logarithms and minima of (quasi-) concave functions. The results thus follow by applying Lemma 1 above and Lemma A5, Parts 2 and 3, in Appendix B. ☐

**Corollary** **1.** *Consider S as a function of $P=(P_{s},P_{r})$. Then $R_{CS}\left(\rho ,S\left(P\right)\right)$ is quasi-concave in $\left(\rho^{2},P\right)$.*

**Proof.**  The proof follows from the proof of Theorem 1 and because S is a linear function of P. ☐

**Figure 3:**
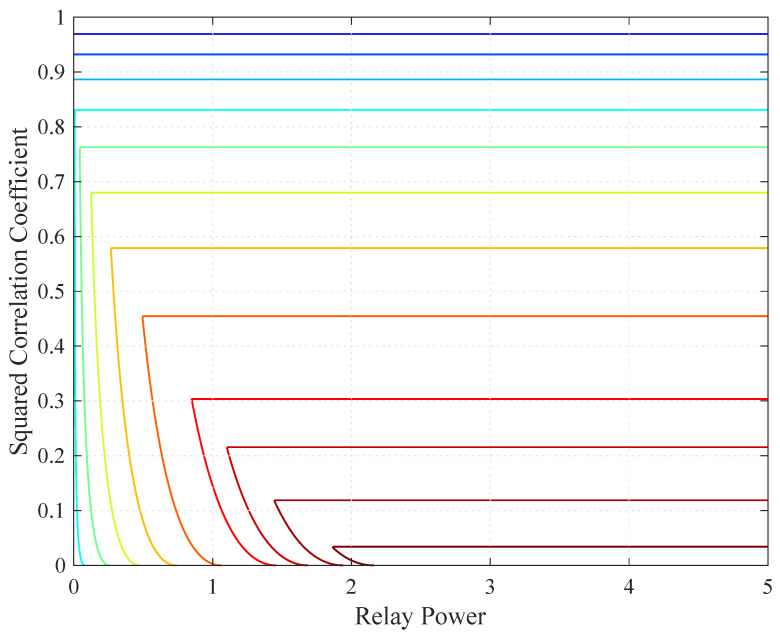
Contour plot of $R_{CS}\left(\rho ,S\left(P\right)\right)$ when $P_{s}=1$.

To illustrate the quasi-concavity, consider one relay and the channel gains $a_{s,r}=5/2$, $a_{s,1}=1$, and $a_{r,1}=5/3$. This scenario corresponds to the geometry in Section 5.1 with r=0.4. Figure 3 shows a contour plot of $R_{CS}\left(\rho ,S\left(P\right)\right)$ when $P_{s}=1$. Observe that the contour lines form convex regions, as predicted by Corollary 1.

### 3.2. DF Rate

Consider the functions

(24) $$\begin{array}{c} g_{j}^{*}\left(\rho ,S\right)=\left(1-\rho^{2}\right){\mathrm{SNR}}_{s,r} \end{array}$$

(25) $$\begin{array}{c} R_{DF}\left(\rho ,S\right)=\min_{1\leq j\leq N}\min\left(C\left(f_{j}\left(\rho ,S\right)\right),C\left(g_{j}^{*}\left(\rho ,S\right)\right)\right). \end{array}$$

As above, we restrict attention to 0≤ρ≤1 and positive S.

**Theorem** **2.** *$R_{DF}\left(\rho ,S\right)$ is concave in ρ, concave in S, and quasi-concave in $\left(\rho^{2},S\right)$.*

**Proof.**  The proof is similar to that of Theorem 1. ☐

**Corollary** **2.** *$R_{DF}\left(\rho ,S\left(P\right)\right)$ is quasi-concave in $\left(\rho^{2},P\right)$.*

**Proof.**  See the proof of Corollary 1. ☐

### 3.3. DT Rate

The DT rate (13) is clearly concave in S and P.

### 3.4. QF Rate

Consider the functions

(26) $$\begin{array}{c} h_{j}\left(S\right)={\mathrm{SNR}}_{s,j}+\frac{{\mathrm{SNR}}_{r,j}{\mathrm{SNR}}_{s,r}}{{\mathrm{SNR}}_{s,j}+{\mathrm{SNR}}_{r,j}+{\mathrm{SNR}}_{s,r}+1} \end{array}$$

(27) $$\begin{array}{c} {\tilde{R}}_{QF}\left(S\right)=\min_{1\leq j\leq N}C\left(h_{j}\left(S\right)\right). \end{array}$$

We establish the following results. We restrict attention to non-negative S.

**Lemma** **2.** *$h_{j}\left(S\right)$ is quasi-concave in $\left({\mathrm{SNR}}_{r,j},{\mathrm{SNR}}_{s,r}\right)$.*

**Proof.**  Substitute $a={\mathrm{SNR}}_{r,j},b={\mathrm{SNR}}_{s,r},k={\mathrm{SNR}}_{s,j}+1$ into the second function of Lemma A6 in Appendix B, and apply Lemma A5, Part 1. ☐

**Theorem** **3.** *${\tilde{R}}_{QF}\left(S\right)$ is quasi-concave in S if the ${\mathrm{SNR}}_{s,j}$, j=1,2,…,n, are held fixed.*

**Proof.**  Apply Lemma 2 above and Lemma A5, Parts 2 and 3, in Appendix B. ☐

## 4. Quasi-Concavity in Relay Position

Suppose the channel gain for the node pair (i,j) is

(28) $$\begin{array}{c} a_{i,j}=\sqrt{\xi_{i,j}}/D_{i,j}^{\alpha /2} \end{array}$$

where $\xi_{i,j}$ is a “fading” gain, $D_{i,j}=∥i-j∥$ is the Euclidean distance between the positions i and j of nodes *i* and *j*, respectively, and α≥2 is a path-loss exponent. We thus have

$$\begin{array}{c} {\mathrm{SNR}}_{i,j}=\frac{\xi_{i,j}P_{i}}{D_{i,j}^{\alpha }}=\frac{\xi_{i,j}P_{i}}{{∥i-j∥}^{\alpha }}. \end{array}$$

We establish quasi-concavity results in $\rho^{2}$ and r, where r is the position of the relay node.

### 4.1. CS Rate

Consider the functions (19)–(21) but relabeled as $f_{j}\left(\rho ,r\right)$, $g_{j}\left(\rho ,r\right)$, and $R_{CS}\left(\rho ,r\right)$ to emphasize the dependence on the considered parameters. We again consider 0≤ρ≤1 and positive S.

**Lemma** **3.** *$f_{j}\left(\rho ,r\right)$ and $g_{j}\left(\rho ,r\right)$ are quasi-concave in r for fixed ρ. Furthermore, $f_{j}\left(\rho ,r\right)$ is quasi-concave in $\left(\rho^{2},r\right)$.*

**Proof.**  Consider the functions

(29) $$\begin{array}{c} {\tilde{f}}_{j}\left(\rho ,D^{\alpha }\right)=\frac{\xi_{s,j}P_{s}}{D_{s,j}^{\alpha }}+\frac{\xi_{r,j}P_{r}}{D^{\alpha }}+2\rho \sqrt{\frac{\xi_{s,j}P_{s}}{D_{s,j}^{\alpha }}\frac{\xi_{r,j}P_{r}}{D^{\alpha }}} \end{array}$$

(30) $$\begin{array}{c} {\tilde{g}}_{j}\left(\rho ,D^{\alpha }\right)=\left(1-\rho^{2}\right)\left(\frac{\xi_{s,j}P_{s}}{D_{s,j}^{\alpha }}+\frac{\xi_{s,r}P_{s}}{D^{\alpha }}\right) \end{array}$$

which are quasi-linear in $D^{\alpha }$ for fixed ρ since they are decreasing in $D^{\alpha }$. However, $D_{r,j}^{\alpha }$ is a convex function of r for α≥1, and thus Lemma A5, Part 5, in Appendix B establishes that $f_{j}\left(\rho ,r\right)$ is quasi-concave in r for fixed ρ. Similarly, $D_{s,r}^{\alpha }$ is a convex function of r for α≥1, and we find that $g_{j}\left(\rho ,r\right)$ is quasi-concave in r for fixed ρ.

Next, substitute $a=D^{\alpha }$ and $b=\rho^{2}$ into the third function of Lemma A6, and use Lemma A5, Part 1, to show that ${\tilde{f}}_{j}\left(\rho ,D^{\alpha }\right)$ is quasi-concave in $\left(\rho^{2},D^{\alpha }\right)$. However, ${\tilde{f}}_{j}$ is decreasing in $D^{\alpha }$ and $D_{r,j}^{\alpha }$ is convex in r, so Lemma A5, Part 5, establishes that $f_{j}\left(\rho ,r\right)$ is quasi-concave in $\left(\rho^{2},r\right)$. ☐

Unfortunately, ${\tilde{g}}_{j}$ is quasi-*convex* (and not quasi-concave) in $\left(\rho^{2},D^{\alpha }\right)$. To see this, substitute $a=D^{\alpha }$ and $b=\rho^{2}$ into the fourth function of Lemma A6. Quasi-concavity would have been useful since it would have permitted using Lemma A5, Parts 2 and 4, to establish the quasi-concavity of

(31) $$\begin{array}{c} R_{CS}\left(r\right)=\max_{\rho }\left[\min_{1\leq j\leq N}\min\left(C\left(f_{j}\left(\rho ,r\right)\right),C\left(g_{j}\left(\rho ,r\right)\right)\right)\right]. \end{array}$$

However, we have been unable to prove this, and our numerical results suggest that $R_{CS}\left(\rho ,r\right)$ is not quasi-concave in $\left(\rho^{2},r\right)$. Nevertheless, Lemma 3 suffices to establish an intermediate result which is useful in Section 5 when we study ρ=0.

**Theorem** **4.** *$R_{CS}\left(\rho ,r\right)$ is quasi-concave in r for fixed ρ, 0≤ρ≤1.*

**Proof.**  $R_{CS}\left(\rho ,r\right)$ is the minimum of functions that are quasi-concave in r. Lemma A5, Part 2, thus establishes the theorem. ☐

### 4.2. DF Rate

The quasi-convexity of ${\tilde{g}}_{j}\left(\rho ,D^{\alpha }\right)$ relaxes for the DF rate (25). Consider the negative of the fourth function of Lemma A6 in Appendix B with $k_{1}=0$:

(32) $$\begin{array}{c} f\left(a,b\right)=\left(1-b\right)k_{2}/a. \end{array}$$

This function is quasi-linear in (a,b) since both its superlevel and sublevel sets are convex. This result implies the following theorem. We again consider the functions (24)–(25) but relabeled as $g_{j}^{*}\left(\rho ,r\right)$ and $R_{DF}\left(\rho ,r\right)$. We further define

(33) $$\begin{array}{c} {\tilde{g}}_{j}^{*}\left(\rho ,D^{\alpha }\right)=\left(1-\rho^{2}\right)\frac{\xi_{s,r}P_{s}}{D^{\alpha }} \end{array}$$

(34) $$\begin{array}{c} R_{DF}\left(r\right)=\max_{\rho }\left[\min_{1\leq j\leq N}\min\left(C\left(f_{j}\left(\rho ,r\right)\right),C\left(g_{j}^{*}\left(\rho ,r\right)\right)\right)\right]. \end{array}$$

As above, we consider 0≤ρ≤1 and positive S.

**Theorem** **5.** *$R_{DF}\left(\rho ,r\right)$ is quasi-concave in $\left(\rho^{2},r\right)$, and $R_{DF}\left(r\right)$ is quasi-concave in r.*

**Proof.**  ${\tilde{g}}_{j}^{*}\left(\rho ,D^{\alpha }\right)$ is quasi-linear in $\left(\rho^{2},D^{\alpha }\right)$ and decreasing in $D^{\alpha }$. Furthermore, $D_{s,r}^{\alpha }$ is convex in r, and thus Lemma A5, Part 5, in Appendix B establishes that $g_{j}^{*}\left(\rho ,r\right)$ is quasi-concave in $\left(\rho^{2},r\right)$. $R_{DF}\left(\rho ,r\right)$ is therefore quasi-concave in r, as it is the minimum of quasi-concave functions (see Lemma A5, Part 2). Furthermore, $R_{DF}\left(r\right)$ is concave in r by Lemma A5, Part 4. ☐

## 5. DF Performance

This section presents numerical results for the DF strategy and compares them to results from [7,8,9]. We consider 1-, 2-, and 3-dimensional MRCs with different numbers *N* of destination nodes. For simplicity, we consider the low SNR or broadband regime where

(35) $$\begin{array}{c} C\left(\mathrm{SNR}\right)=\frac{1}{2}\log\left(1+\mathrm{SNR}\right)\to \frac{1}{2}\mathrm{SNR}. \end{array}$$

In other words, we consider the CS and DF rates without the logarithms. This approach is valid not only in the limit of low SNR, but more generally because we proved our quasi-concavity results without taking logarithms. Furthermore, in the low SNR regime the rates of full-duplex and half-duplex transmission are the same under a block power constraint.

We choose $P_{s}=P_{r}=P=1$, α=2, and $\xi_{u,v}=1$ for all node pairs (u,v). We study both coherent transmission where ρ is optimized and non-coherent transmission with ρ=0. The rates are in nats/channel use. Alternatively, suppose we use sync pulses sampled at 2W samples per second, where *W* is the (one-sided) signal bandwidth. Suppose further that the (one-sided) noise power spectral density is 1 Watt/Hz. Then at low SNR the rates in nats/channel use are the same as the rates in nats/sec.

### 5.1. One Dimension

**Figure 4:**
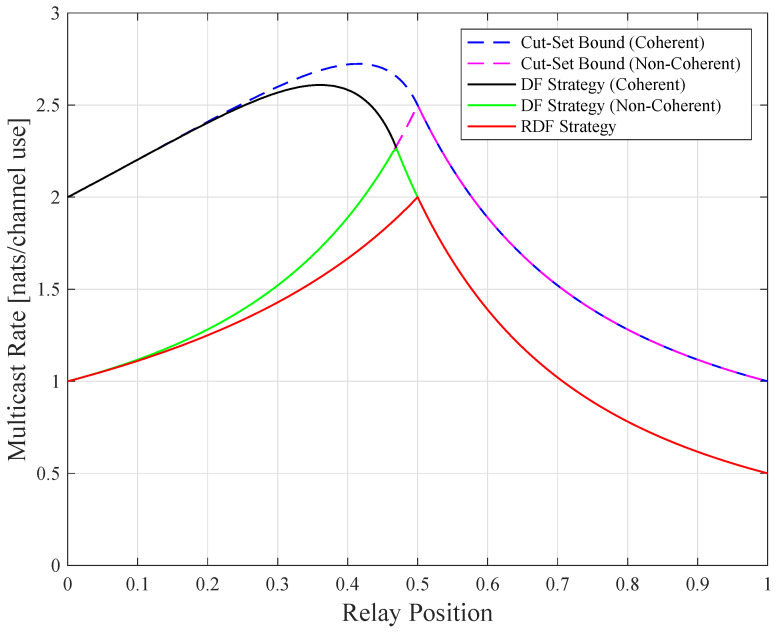
Relay channel rates for low signal-to-noise ratio (SNR) and P=1.

Consider a relay channel (N=1) where the source is at the origin (s=0) and the destination is at point 1 (1=1). Figure 4 shows the low SNR CS rates, DF rates, and the routing-based DF (RDF) rates developed in [7], which are given by

(36) $$\begin{array}{c} R_{CS}\to \frac{1}{2}\min\left(\frac{\xi_{s,1}P_{s}}{{∥s-1∥}^{\alpha }}+\frac{\xi_{r,1}P_{r}}{{∥r-1∥}^{\alpha }}+\frac{2\rho \sqrt{\xi_{s,1}\xi_{r,1}P_{s}P_{r}}}{{∥s-1∥}^{\alpha /2}{∥r-1∥}^{\alpha /2}},\;\left(1-\rho^{2}\right)\left(\frac{\xi_{s,1}P_{s}}{{∥s-1∥}^{\alpha }}+\frac{\xi_{s,r}P_{s}}{{∥s-r∥}^{\alpha }}\right)\right) \end{array}$$

(37) $$\begin{array}{c} R_{DF}\to \frac{1}{2}\min\left(\frac{\xi_{s,1}P_{s}}{{∥s-1∥}^{\alpha }}+\frac{\xi_{r,1}P_{r}}{{∥r-1∥}^{\alpha }}+\frac{2\rho \sqrt{\xi_{s,1}\xi_{r,1}P_{s}P_{r}}}{{∥s-1∥}^{\alpha /2}{∥r-1∥}^{\alpha /2}},\;\left(1-\rho^{2}\right)\frac{\xi_{s,r}P_{s}}{{∥s-r∥}^{\alpha }}\right) \end{array}$$

(38) $$\begin{array}{c} R_{RDF}\to \max_{0\leq \beta \leq 1}\frac{1}{2}\left[\min\left(\frac{\xi_{r,1}P_{r}}{{∥r-1∥}^{\alpha }},\;\frac{\beta \;\xi_{s,r}P_{s}}{{∥s-r∥}^{\alpha }}\right)+\frac{\left(1-\beta \right)\;\xi_{s,1}P_{s}}{{∥s-1∥}^{\alpha }}\right]. \end{array}$$

Observe that all curves are quasi-concave (but not concave) in r. Theorems 4 and 5 predict the quasi-concavity for all curves except for the coherent CS rates. Observe also that the curves for the coherent and non-coherent rates merge for relay positions exceeding a certain value (r=0.5 and r≈0.47 for the respective CS and DF rates). The reason for this behavior is that ρ=0 is optimal for the coherent CS and DF rates beyond these positions, see the ρ curve in [1] (Figure 16). Furthermore, the non-coherent CS rates coincide with the non-coherent DF rates for a large range of r.

The best relay positions for the two strategies are different. For example, r=0.5 maximizes $R_{RDF}$ while the r maximizing $R_{DF}$ is closer to the source. This is because when the source transmits, the relay and the destination listen, and the destination “collects” information. The relay can thus be positioned closer to the source while maintaining the same information rate from the source to the relay, and from the source-relay pair to the destination. At the optimal positions, we compute $R_{DF}\approx 2.26P$ nats/sec and $R_{RDF}=2P$ nats/sec, so the DF gain is ≈13%.

**Figure 5:**
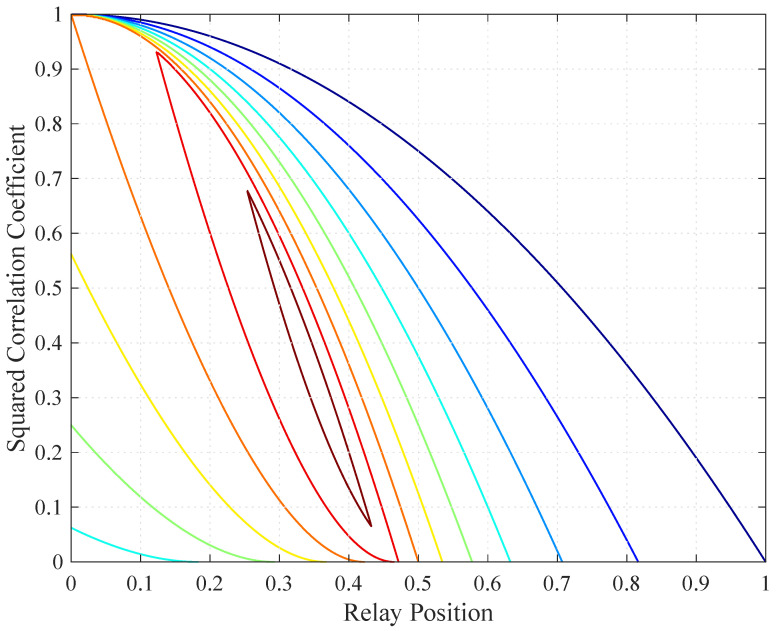
Contour plot of $R_{DF}\left(\rho ,r\right)$ in (37).

Finally, we illustrate that $R_{DF}\left(\rho ,r\right)$ is quasi-concave in $\left(\rho^{2},r\right)$ in Figure 5. The contour lines form convex regions, as predicted by Theorem 5.

### 5.2. Two Dimensions

**Figure 6:**
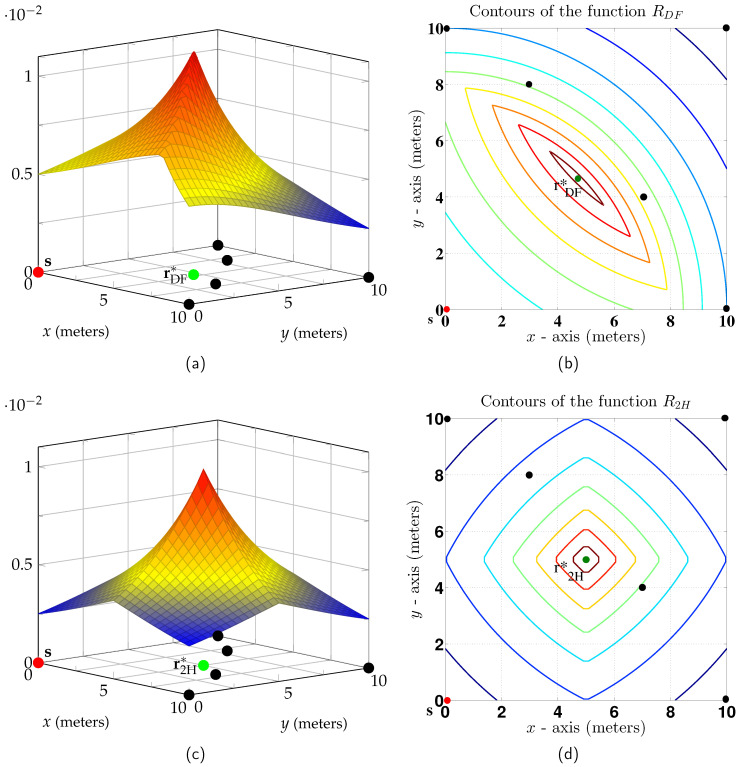
(**a**) $R_{DF}$ for N=5; (**b**) $R_{DF}$ contour plot; (**c**) $R_{2H}$ for the same network; (**d**) $R_{2H}$ contour plot.

Consider N=5 destinations positioned on a square in the two-dimensional Euclidean plane with the source node at the origin. Figure 6a plots the node positions as circles, and the non-coherent $R_{DF}$ as a function of the relay position. The best relay position is shown by a circle labeled $r_{\mathrm{DF}}^{*}$ and the corresponding rate is $R_{DF}\approx 0.011P$ nats/sec. Figure 6c plots the low SNR two-hop rate

(39) $$\begin{array}{c} R_{2H}\to \min_{1\leq j\leq 5}\frac{1}{2}\min\left(\frac{\xi_{s,r}P_{s}}{{∥s-r∥}^{\alpha }},\frac{\xi_{r,j}P_{r}}{{∥r-j∥}^{\alpha }}\right) \end{array}$$

as a function of the relay position. The best relay position is shown by a circle labeled $r_{2H}^{*}$ and the corresponding two-hop rate is $R_{2H}=0.01P$ nats/sec. The non-coherent DF gain is thus ≈10%.

Figure 6b,d shows contour plots for $R_{DF}$ and $R_{2H}$. The contours form convex regions, as predicted by Theorem 5. Again, the relay position maximizing $R_{DF}$ lies closer to the source than the relay position maximizing $R_{2H}$.

### 5.3. Three Dimensions

**Figure 7:**
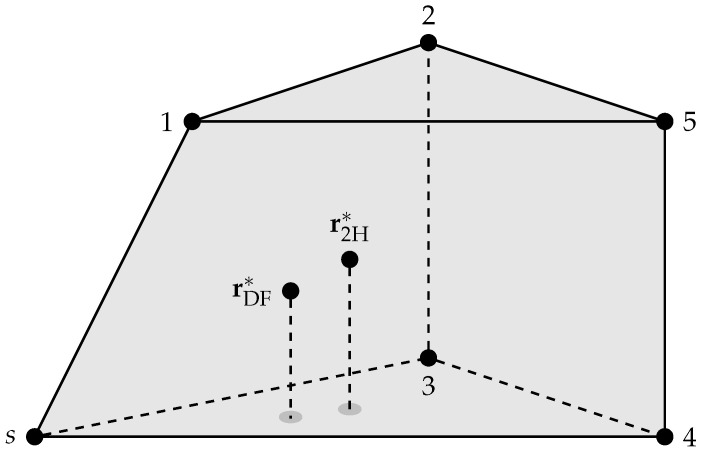
N=5 destination geometry in three dimensions.

Consider N=5 destinations positioned in 3-dimensional Euclidean space as in Figure 7. The figure also shows the convex hull (a polyhedron) of the points. The points $r_{\mathrm{DF}}^{*}$ and $r_{2H}^{*}$ denote the relay positions that maximize the non-coherent $R_{DF}$ and $R_{2H}$, respectively. We remark that $r_{\mathrm{DF}}^{*}$ and $r_{2H}^{*}$ remain unchanged if more destinations are positioned inside the polyhedron. This is because the points in the polyhedron receive at least the same rate as the worst of the five nodes at the corner points.

## 6. Discussion

### 6.1. Complex AWGN Channels

For complex-alphabet AWGN channels, we could replace (5) and (6) by adding phases $\phi_{i,j}$ for i=s,r and j=1,2,…,N as follows:

(40) $$\begin{array}{cc} Y_{r} & =a_{s,r}\;e^{j\phi_{s,r}}\;X_{s}+Z_{r} \end{array}$$

(41) $$\begin{array}{cc} Y_{j} & =a_{s,j}\;e^{j\phi_{s,j}}\;X_{s}+a_{r,j}\;e^{j\phi_{r,j}}\;X_{r}+Z_{j} \end{array}$$

where the noise variables $Z_{r}$ and $Z_{j}$, j=1,2,…,N, are independent, identically distributed, circularly symmetric, complex, Gaussian random variables with zero mean and unit variance. The distance dependence of $a_{i,j}$ can be chosen as in (28) and the phase dependence as

(42) $$\begin{array}{c} \phi_{i,j}=2\pi D_{i,j}/\lambda \end{array}$$

where $\lambda =c/f_{o}$ is the wavelength, *c* is the speed of light, and $f_{o}$ is the carrier frequency.

For example, the DF rate (14), normalized by the number of real dimensions, is

(43) $$\begin{array}{c} R_{DF}=\max_{\rho }\left[\min_{1\leq j\leq N}\min\left(C({\mathrm{SNR}}_{s,j}+{\mathrm{SNR}}_{r,j}+\right.\right.\\ \left.\left.\left.\;2ℜ\left\{\rho e^{j(\phi_{s,j}-\phi_{r,j})}\right\}\sqrt{{\mathrm{SNR}}_{s,j}{\mathrm{SNR}}_{r,j}}\right),C((1-{\left|\rho \right|}^{2}){\mathrm{SNR}}_{s,r})\right)\right] \end{array}$$

where the complex correlation coefficient ρ satisfies 0≤|ρ|≤1. Observe that for a classic relay channel, with N=1 destination, one can choose ρ to make $ℜ\left\{\rho e^{j(\phi_{s,1}-\phi_{r,1})}\right\}$ real and non-negative, as for real alphabet AWGN channels. However, for N≥2 one must choose complex ρ in general. Furthermore, the quasi-concavity in r will not be valid in general because the phases $\phi_{r,j}$ change with r, and we cannot optimize ρ for each destination node separately. However, we remark that this effect is “local" in the sense that for large carrier frequencies the phase variations are sensitive to changes in r. A pragmatic approach would then be to optimize r for non-coherent transmission (ρ=0) even if beamforming is permitted. Furthermore, if the channel exhibits random phase variations, then the best approach is to choose ρ=0 (see [1], Figure 18) in which case we have quasi-concavity for both the CS and DF rates. Finally, we remark that it might be interesting to consider quasi-concavity in the correlation coefficients for problems where the source and relay have sufficiently many antennas to overcome the problem outlined above.

### 6.2. Sum-Power Constraint

For some applications, it is interesting to consider a *sum*-power constraint

(44) $$\begin{array}{c} E\left[\sum_{k=1}^{n}{\left|X\right|}_{s,k}^{2}+{\left|X\right|}_{r,k}^{2}\right]\leq nP_{T}. \end{array}$$

As is usually done, we set $P_{r}=P_{T}-P_{s}$ and consider $P_{s}$, $0\leq P_{s}\leq P_{T}$ as a new optimization parameter. One might now hope that $R_{CS}\left(P_{s},r\right)$ or $R_{DF}\left(P_{s},r\right)$ are quasi-concave in $\left(P_{s},r\right)$ for fixed ρ, or at least for ρ=0. Unfortunately, we have found counterexamples that show this is not the case. The rate functions do seem to have interesting properties, however, and these deserve further exploration.

## 7. Conclusions

Various quasi-concavity results were established for AWGN MRCs. In particular, the CS rates are quasi-concave in the relay position for a fixed correlation coefficient (Theorem 4) and the DF rates are quasi-concave in the relay position (Theorem 5).

## Appendix A. Covariance Matrices and Concavity

The covariance matrix of a real-valued random column vector V is

(A1) $$\begin{array}{c} Q_{V}=E\left[\left(V-E\left[V\right]\right){\left(V-E\left[V\right]\right)}^{T}\right]. \end{array}$$

A useful property of covariance matrices is as follows (see [14], p. 684). If $Q_{V}^{*}$ is a principal minor of $Q_{V}$, then the following function is concave in $Q_{V}$:

(A2) $$\begin{array}{c} f\left(Q_{V}\right)=\log\frac{\det Q_{V}}{\det Q_{V}^{*}}. \end{array}$$

## Appendix B. Concave and Quasi-Concave Functions

We review results on quasi-concavity, and then establish quasi-concavity for several functions.

### Appendix B.1. Definitions

Consider the following sets. The *domain* $D_{f}$ of a real-valued function $f:R^{n}\to R$ is the set of arguments for which *f* is defined. The *hypograph* and *hypergraph* of *f* are the respective

(A3) $$\begin{array}{c} H_{f}=\left\{\left(x,y\right)\left|y\leq f(x)\right.\right\},\;{\hat{H}}_{f}=\left\{\left(x,y\right)\left|y\geq f(x)\right.\right\}. \end{array}$$

The *superlevel* and *sublevel* sets of *f* with respect to β∈R are the respective

(A4) $$\begin{array}{c} S_{f,\beta }=\left\{x|f\left(x\right)\geq \beta \right\},\;{\hat{S}}_{f,\beta }=\left\{x|f\left(x\right)\leq \beta \right\}. \end{array}$$

Concave and quasi-concave functions can be defined via the convexity of these sets. Recall that a set S, $S\subseteq R^{n}$, is *convex* if for any two points $x_{1}$ and $x_{2}$ in S and for any λ satisfying 0≤λ≤1 we have

(A5) $$\begin{array}{c} \lambda x_{1}+\bar{\lambda }x_{2}\in S \end{array}$$

where $\bar{\lambda }=1-\lambda$. Suppose that $D_{f}$ is convex. The function *f* is *concave* over $D_{f}$ if and only if its hypograph $H_{f}$ is convex. Similarly, *f* is *convex* over $D_{f}$ if and only if ${\hat{H}}_{f}$ is convex. The function *f* is *quasi-concave* over $D_{f}$ if and only if all its superlevel sets are convex, and *f* is *quasi-convex* over $D_{f}$ if and only if all its sublevel sets are convex. A function that is quasi-convex and quasi-concave is called *quasi-linear*. For example, any non-increasing or non-decreasing function is quasi-linear.

### Appendix B.2. Basic Properties

Two properties of concave and quasi-concave functions are as follows; these properties are often used as the definitions of such functions. Similar properties exist for convex and quasi-convex functions.

**Lemma** **A1.**
*The function f is concave if and only if*

(A6) $$\begin{array}{c} f\left(\lambda x_{1}+\bar{\lambda }x_{2}\right)\geq \lambda f\left(x_{1}\right)+\bar{\lambda }f\left(x_{2}\right) \end{array}$$

*for all $x_{1}$ and $x_{2}$ in $D_{f}$ and for all 0≤λ≤1.*

**Lemma** **A2.**
*The function f is quasi-concave if and only if*

(A7) $$\begin{array}{c} f\left(\lambda x_{1}+\bar{\lambda }x_{2}\right)\geq \min\left(f\left(x_{1}\right),f\left(x_{2}\right)\right) \end{array}$$

*for all $x_{1}$ and $x_{2}$ in $D_{f}$ and for all 0≤λ≤1.*

The next two properties assume that *f* is twice differentiable and that $D_{f}$ is convex. Let $H_{f}\left(x\right)$ and $B_{f}\left(x\right)$ be the respective Hessian and bordered Hessian of *f* at x.

**Lemma** **A3.**
*(see [15], Section 3.1.4) f is concave if and only if $H_{f}\left(x\right)$ is negative semidefinite for all $x\in D_{f}$.*

**Lemma** **A4.**
*(see [16], p. 771) f is quasi-concave on the open and convex set $D_{f}$ if the determinants $D_{2},D_{3},\ldots ,D_{n}$ of the respective second to nth leading principal minors of $B_{f}\left(x\right)$ satisfy ${\left(-1\right)}^{k}D_{k}<0$ for k=2,3,…,n and for all $x\in D_{f}$.*

### Appendix B.3. Compositions Preserving Quasi-Concavity

The following compositions preserve quasi-concavity.

**Lemma** **A5.**
*Suppose f and $f_{i}$, 1≤i≤n, are quasi-concave, then so are the functions*

1. *$h=k_{1}f+k_{2}$, where $k_{1}\geq 0$ and $k_{2}\in R$;*
2. *$h=\min_{1\leq i\leq n}f_{i}$;*
3. *h=g∘f where f is quasi-concave and g is non-decreasing;*
4. *$h\left(a\right)=\sup_{b\in B}f\left(a,b\right)$ where B is a convex set;*
5. *h(a,b)=f(g(a),b) where g is convex and $f(\tilde{a},b)$ is non-increasing in $\tilde{a}$ for fixed b.*

**Proof.** Properties 1)–4) are standard (see [15], Section 3.4). For property 5), observe that

(A8) $$\begin{array}{c} h(\lambda a_{1}+\bar{\lambda }a_{2},\lambda b_{1}+\bar{\lambda }b_{2})\\ =f(g\left(\lambda a_{1}+\bar{\lambda }a_{2}\right),\lambda b_{1}+\bar{\lambda }b_{2})\\ \overset{\left(a\right)}{\geq }f\left(\lambda g\left(a_{1}\right)+\bar{\lambda }g\left(a_{2}\right),\lambda b_{1}+\bar{\lambda }b_{2}\right)\\ \overset{\left(b\right)}{\geq }\min\left(f\left(g\left(a_{1}\right),b_{1}\right),f\left(g\left(a_{2}\right),b_{2}\right)\right) \end{array}$$

where (a) follows because $g\left(\lambda a_{1}+\bar{\lambda }a_{2}\right)\leq \lambda g\left(a_{1}\right)+\bar{\lambda }g\left(a_{2}\right)$ and $f(\tilde{a},b)$ is non-increasing in $\tilde{a}$. Step (b) follows because *f* is quasi-concave. ☐

### Appendix B.4. Examples of Quasi-Concave Functions

We establish quasi-concavity for several useful functions.

**Lemma** **A6.**
*The following functions are quasi-concave for x=(ab) with non-negative entries.*

1. f(x)=ab
2. *$f\left(x\right)=\frac{ab}{a+b+k}$ for a positive constant k*
3. *$f\left(x\right)=k_{1}/a+2\sqrt{k_{2}b/a}$ for positive constants $k_{1},k_{2}$*
4. *$f\left(x\right)=-\left(1-b\right)\left(k_{1}+k_{2}/a\right)$ for positive constants $k_{1},k_{2}$, and b≤1*

*Furthermore, the following function is quasi-concave for x=(abc) with non-negative entries.*

5. $f\left(x\right)=a+b+2\sqrt{abc}$

**Proof.** We consider positive x, and we use bordered Hessians $B_{f}\left(x\right)$ and the derivatives $D_{k}$ of their *k*th leading principal minors, k=2,3,…,n. The results extend to non-negative x by using continuity at zero values, except for the third and fourth functions where a=0 makes the functions undefined.

1. We have $D_{2}<0$ and $D_{3}>0$ for
  $$\begin{array}{cc} B_{f}\left(x\right)= & \left(\begin{array}{ccc} 0 & b & a\\ b & 0 & 1\\ a & 1 & 0 \end{array}\right). \end{array}$$
2. We have $D_{2}<0$ and $D_{3}>0$ for
  $$\begin{array}{cc} B_{f}\left(x\right)= & \left(\begin{array}{ccc} 0 & \frac{b(b+k)}{{\left(a+b+k\right)}^{2}} & \frac{a(a+k)}{{\left(a+b+k\right)}^{2}}\\ \frac{b(b+k)}{{\left(a+b+k\right)}^{2}} & \frac{-2b(b+k)}{{\left(a+b+k\right)}^{3}} & \frac{2ab+(a+b+k)k}{{\left(a+b+k\right)}^{3}}\\ \frac{a(a+k)}{{\left(a+b+k\right)}^{2}} & \frac{2ab+(a+b+k)k}{{\left(a+b+k\right)}^{3}} & \frac{-2a(a+k)}{{\left(a+b+k\right)}^{3}} \end{array}\right). \end{array}$$
3. We have $D_{2}<0$ and $D_{3}>0$ for
  $$\begin{array}{cc} B_{f}\left(x\right)= & \left(\begin{array}{ccc} 0 & -\frac{k_{1}+\sqrt{k_{2}ab}}{a^{2}} & \sqrt{\frac{k_{2}}{ab}}\\ -\frac{k_{1}+\sqrt{k_{2}ab}}{a^{2}} & \frac{4k_{1}+3\sqrt{k_{2}ab}}{2a^{3}} & -\frac{\sqrt{k_{2}}}{2a^{3/2}\sqrt{b}}\\ \sqrt{\frac{k_{2}}{ab}} & -\frac{\sqrt{k_{2}}}{2a^{3/2}\sqrt{b}} & -\frac{\sqrt{k_{2}}}{2b^{3/2}\sqrt{a}} \end{array}\right). \end{array}$$
4. If b≤1, we have $D_{2}<0$ and $D_{3}>0$ for
  $$\begin{array}{cc} B_{f}\left(x\right)= & \left(\begin{array}{ccc} 0 & \frac{\left(1-b\right)k_{2}}{a^{2}} & k_{1}+\frac{k_{2}}{a}\\ \frac{\left(1-b\right)k_{2}}{a^{2}} & -\frac{2\left(1-b\right)k_{2}}{a^{3}} & -\frac{k_{2}}{a^{2}}\\ k_{1}+\frac{k_{2}}{a} & -\frac{k_{2}}{a^{2}} & 0 \end{array}\right). \end{array}$$
5. We have $D_{2}<0$, $D_{3}>0$ and $D_{4}<0$ for
  (A9) $$\begin{array}{c} B_{f}\left(x\right)=\left(\begin{array}{cccc} 0 & 1+\sqrt{\frac{bc}{a}} & 1+\sqrt{\frac{ac}{b}} & \sqrt{\frac{ab}{c}}\\ 1+\sqrt{\frac{bc}{a}} & -\frac{\sqrt{bc}}{2a^{3/2}} & \frac{1}{2}\sqrt{\frac{c}{ab}} & \frac{1}{2}\sqrt{\frac{b}{ac}}\\ 1+\sqrt{\frac{ac}{b}} & \frac{1}{2}\sqrt{\frac{c}{ab}} & -\frac{\sqrt{ac}}{2b^{3/2}} & \frac{1}{2}\sqrt{\frac{a}{bc}}\\ \sqrt{\frac{ab}{c}} & \frac{1}{2}\sqrt{\frac{b}{ac}} & \frac{1}{2}\sqrt{\frac{a}{bc}} & -\frac{\sqrt{ab}}{2c^{3/2}} \end{array}\right). \end{array}$$

 ☐
